# Thermal Stabilization of Viral Vaccines in Low-Cost Sugar Films

**DOI:** 10.1038/s41598-019-44020-w

**Published:** 2019-05-21

**Authors:** Vincent Leung, Jonathan Mapletoft, Ali Zhang, Amanda Lee, Fatemeh Vahedi, Marianne Chew, Alexandra Szewczyk, Sana Jahanshahi-Anbuhi, Jann Ang, Braeden Cowbrough, Matthew S. Miller, Ali Ashkar, Carlos D. M. Filipe

**Affiliations:** 1 Department of Chemical Engineering, 1280 Main Street West, McMaster University, Hamilton, Ontario L8S 4L7 Canada; 2 Michael G. DeGroote Institute for Infectious Disease Research, McMaster Immunology Research Centre, Department of Biochemistry and Biomedical Sciences, McMaster University, Hamilton, ON Canada; 3 Department of Pathology and Molecular Medicine, McMaster Immunology Research Centre, McMaster University, Rm 4015 Michael DeGroote Centre for Learning and Discovery, 1280 Main Street West, Hamilton, ON L8S 4K1 Canada

**Keywords:** Immunology, Biotechnology

## Abstract

Most currently available vaccines, particularly live vaccines, require the cold chain, as vaccine efficacy can be significantly hampered if they are not stored in a temperature range of 2–8 °C at all times. This necessity places a tremendous financial and logistical burden on vaccination programs, particularly in the developing world. The development of thermally stable vaccines can greatly alleviate this problem and, in turn, increase vaccine accessibility worldwide. In this paper, we detail a simple and cost-effective method for stabilizing live vaccines that uses FDA-approved materials. To this end, we dried enveloped DNA (Herpes Simplex Virus type 2) and RNA (Influenza A virus) viral vaccines in a pullulan and trehalose mixture. The results of these studies showed that the live-attenuated HSV-2 vaccine retained its efficacy for at least 2 months of storage at 40 °C, while the inactivated influenza vaccine was able to retain its immunogenicity for at least 3 months of storage at 40 °C. This work presents a simple approach that allows thermo-sensitive vaccines to be converted into thermo-stable vaccines that do not require refrigeration, thus contributing to the improvement of vaccine deployment throughout the world.

## Introduction

Vaccination is a critical component of global health that saves millions of lives each year. Unfortunately, almost all available vaccines are thermally labile and must be stored at temperatures between 2–8 °C at all times, from production to dispensation, in order to maintain their efficacy^1^. This uninterrupted refrigerated supply chain is known as the “cold chain”, and any failure to maintain it can result in wastage or the administration of ineffective vaccines^2^. The need for the cold chain is one of the major causes of under-vaccination globally, as it presents significant economic and logistic problems for vaccination programs. This problem is especially serious in developing countries and remote areas, which often lack dependable cold chain infrastructure and/or access to reliable electricity^3–5^. These challenges are compounded with rapid global climate change which have significantly increased the spread of infectious diseases such as malaria, dengue fever, and zika^6–9^. Therefore, the development of a versatile thermal stabilization platform for vaccines is a pressing need as it would greatly increase access to, and significantly decrease the costs of, vaccination programs in under-serviced areas.

The need for vaccines that are capable of remaining active outside of the cold chain has been recognized by researchers, who have made considerable efforts to create thermally stable vaccines and/or vaccine carriers. One approach taken has been to engineer vaccines that can maintain their thermal stability without preservative adjuvants. For example, Sun *et al*. demonstrated that it is possible to produce a vaccine with strong thermal stability by attaching an M. tuberculosis epitope to a self-assembling fibril-forming peptide. Using this method, they were able to produce a vaccine that could be stored for 7 days at 45 °C without exhibiting any conformational change^10^. Similarly, Beernink’s group engineered a mutant antigen for a recombinant meningococcal vaccine that increased its thermal tolerance by 21 °C^11,12^, while Campeotto *et al*. were able to increase the thermal tolerance of a malaria-protein vaccine by 10–15 °C by modifying it via the introduction of 18 mutations^13^. Other researchers have attempted to create thermally stable vaccines by modifying viral vectors. In one such study, Stobart *et al*. engineered a respiratory syncytial virus (RSV) that featured enhanced pre-F expression, which resulted in greater immunogenicity and thermal stability than the wild type^14^. However, despite their success, Stobart *et al*. noted that there was still significant loss in titer after 7 days of storage at 37 °C^14^. In a different study, Wang *et al*. used a human enterovirus type 71 vaccine to develop a self-biomineralized virus that could be stored for 1 week at 37 °C^15^. Although these thermally stable vaccines hold some promise, many still have short shelf lives (~7 days) when stored at elevated temperature (>37 °C). Moreover, these challenges are compounded by the fact that, not only is the engineering of new vaccines labor intensive, but all newly developed vaccines must obtain governmental approval before they are deployed.

Another common approach to thermally stabilizing vaccines is through the addition of stabilizing adjuvants. For instance, Pelliccia *et al*. created thermally stable adenoviral vaccine formulations that could maintain their immunogenicity for up to 10 days at 37 °C by adding polyethylene glycol (PEG), gold nanoparticles (AuNP), and sucrose^16^. In addition to the use of stabilizing adjuvants, vaccines are often dried in order to further increase their thermal stability. Prausntz’s group was able to preserve the immunogenicity of inactivated influenza vaccine over a storage period of 4 months at 60 °C by encapsulating it in microneedle patches using different stabilizing adjuvant formulations^17–19^. Hassett *et al*. showed that lyophilized anthrax vaccine maintained its immunogenicity after 16 weeks of storage at 40 °C^20^, and lyophilized recombinant ricin toxin A vaccine retained its stability after being stored for 4 weeks at 40 °C^21^. Chen *et al*.^22^ thermally stabilized formulations of a recombinant hepatitis B vaccine and meningitis A protein-polysacharide conjugate vaccine by spray drying. The spray-dried vaccine formulations were shown to be stable for 24 months at 37 °C. In a different study, Ohtake *et al.*^23^. Preserved the potency of an attenuated salmonella enterica vaccine via foam drying using trehalose, methionine and gelatin as stabilizers. The foam-dried vaccine was stable over a storage period of 12 weeks at 37 °C. The same research group maintained the stability of a live-attenuated measles vaccine for up to 8 weeks at 37 °C by using sugar- and protein-based spray drying formulations^24^. Similarly, Lovalenti *et al*. successfully stabilized live-attenuated influenza vaccines in a sucrose-containing excipient using three drying methods: freeze drying, spray drying, and foam drying. They found that, when the right excipient composition was used, foam drying produced the most thermally stable vaccine, with a shelf life of 4.5 months at 37 °C^25^. Other research has demonstrated that the use of different lyophilized formulations can be used to retain the potency of rotavirus vaccines for up to 20 months at 37 °C. The lyophilized rotavirus vaccine is currently commercially available^26,27^. Alcock *et al*. were able to secure titer retention in adenovirus and modified vaccinia virus Ankara for up to 6 months at 45 °C by drying them onto polypropylene or glass fiber membranes using sucrose and trehalose^28^. Although the above studies have made promising breakthroughs in providing alternatives that do not require the cold chain, their applicability is limited as freeze drying, spray drying, and foam drying all require specialized equipment for sample preparation (freeze dryer, vacuum pumps) and the exposure of vaccines to extreme temperatures or pressure conditions^23^. Moreover, some of the formulations used in these studies require a large number of adjuvants, which can increase the cost and complexity of the vaccine product.

In this study, we document a simple and low-cost method for thermally stabilizing two enveloped viruses: Herpes Simplex Virus type 2 (HSV-2), which is a DNA virus; and influenza A virus (IAV), which is an RNA virus. The proposed method is predicated on drying the viruses in sugar film made from a mixture of pullulan and trehalose. Trehalose is a disaccharide that is commonly used as a cryoprotectant and stabilizing agent^29–34^, while pullulan is a polysaccharide with good film-forming abilities that is used in the food industry to extend the shelf life of food^35–39^. In addition to being readily available and inexpensive, these two compounds are also FDA approved. In our previous studies, we have shown that the joint use of pullulan and trehalose can provide long-term stabilization for enzymes and bacteriophages by protecting them against oxidation and thermal inactivation^40–42^. Vaccines containing live-enveloped viruses were chosen for this study because they are intrinsically more unstable than other types of vaccines^25,28^. Herein, we demonstrate that the *in vitro* infectivity of these live viruses, and the *in vivo* immunogenicity of their corresponding vaccines, can be preserved for up to 3 months at 40 °C by drying them in a pullulan and trehalose mixture.

## Results

### Pullulan and trehalose (PT) film provides thermal protection for HSV-2 *in vitro*

**Figure 1:**
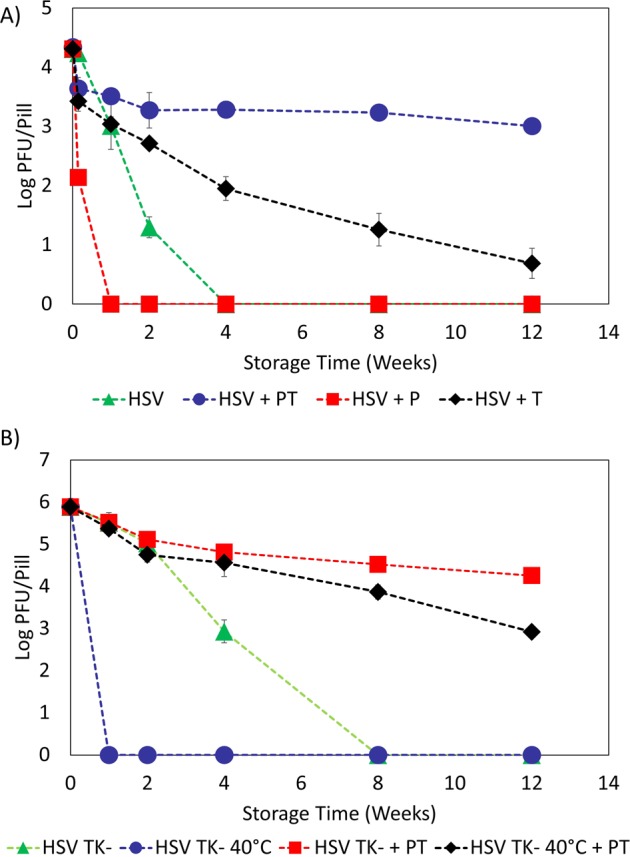
*In-vitro* Thermal Stabilization of HSV-2 and HSV-2 TK^−^: (**A**) Titers of HSV-2 stored in pullulan (P), trehalose (T), and pullulan and trehalose (PT) as a function of storage time at room temperature; **(B)** TK^−^ HSV-2 and TK^−^ HSV-2 + PT titer as a function of storage time at room temperature and at 40 °C. All *in vitro* experiments were performed in triplicate. The error bars represents the standard deviation.

We initially dried HSV-2 (strain 333) in three different drying matrices to determine each one’s effectiveness as a stabilizer. The three matrices used were as follows: (1) 10 wt% pullulan; (2) 0.5 M trehalose; and (3) a mixture of 10 wt% pullulan with 0.5 M trehalose. Each sample had an initial titer of 2 × 10^4^ plaque-forming units (PFU) and was stored at room temperature (~23 °C). Following drying, the titer of each sample was determined at different storage times and the log difference was calculated by comparing the titer at each storage time to the initial titer prior to drying. As shown in Fig. 1(A), HSV-2 dried in the solution containing 10 wt% pullulan and 0.5 M trehalose (Matrix 3) most effectively maintained its viral titer, only losing 2.3 log PFU/film after 12 weeks of storage. In comparison, HSV-2 dried in the matrix containing only 0.5 M trehalose (Matrix 2) showed a titer loss of 3.6 log PFU/film after 12 weeks, while the HSV-2 that had been dried in the matrix containing 10 wt% pullulan (Matrix 1) was completely inactive after 7 days. Furthermore, the HSV-2 sample that had been stored in PBS buffer was found to be completely inactive within 28 days.

The results also show that, on its own, pullulan offers little protection against desiccation, as HSV-2 dried in pullulan registered a titer loss of 2.2 log PFU/film during the drying process. Conversely, the HSV-2 samples that had been dried in trehalose alone and the pullulan/trehalose (PT) mixture showed respective titer losses of 0.9 log PFU/film and 0.7 PFU/film during the drying process. Moreover, drying HSV-2 in trehalose alone did not offer long-term stability. After four weeks, HSV-2 dried in trehalose alone had a titer loss of 2.4 log PFU/film, while HSV-2 that had been dried in PT film had a titer loss of only 1.0 log PFU/film. Furthermore, HSV-2 in PT film demonstrated good stability after two weeks of storage. Between Week 2 and Week 12, there was only a loss of 0.3 log PFU/film. In comparison, the HSV-2 samples dried in trehalose registered a titer loss of 2.0 log PFU/film between Week 2 and Week 12. These results demonstrate pullulan’s and trehalose’s synergistic effects as a stabilizing matrix, and they support the findings of our previous work wherein we stabilized bacteriophages in sugar films^42^.

In order to further analyze the ability of PT film to provide thermal stability at elevated temperatures, we dried samples of live-attenuated thymidine kinase-deficient strain of HSV-2 which has been investigated as a vaccine candidate^43^ (HSV-2 TK^−^, initial titer: 10^6^ PFU) in 10 wt% pullulan and 0.5 M trehalose and stored them at room temperature and at 40 °C. The titers of the samples in the PT film were determined for each temperature condition at different time points over a 12 week period and compared to corresponding samples of HSV-2 TK^−^ that had not been dried in PT film. As Fig. 1(B) shows, HSV-2 TK^−^ stored in PT film had a titer loss of 1.6 log PFU/film when stored at room temperature and a titer loss of 3.0 log PFU/film when stored at 40 °C. In contrast, HSV-2 TK^−^ without pullulan and trehalose was completely inactive within 8 weeks when stored at room temperature and within 1 week when stored at 40 °C. Moreover, during the first 4 weeks, the storage temperature did not significantly affect the stability of HSV-2 TK^−^ in PT films. However, at 8 weeks and 12 weeks, the HSV-2 TK^−^ in PT film was more stable at room temperature than at 40 °C. Lastly, it is important to note that the initial titer for the HSV-2 TK^−^ experiment was much higher (10^6^ PFU) than for the HSV-2 experiments (2 × 10^4^). The ratio between the virus concentration and pullulan/trehalose concentration may play a role in the stabilization effectiveness, however, this ratio was not explored in this study. Our previous study showed that similar volumes of pullulan/trehalose can provide long-term stabilization for up to 10^9^ PFU of bacteriophage. Although the bacteriophage study suggests that PT films may be able to stabilize higher concentrations of viruses, however, since the stability of different viruses can vary widely, further study is required to determine the stabilization effectiveness of pullulan/trehalose at different concentrations of HSV-2. Overall, these *in vitro* results demonstrate that PT films offer significant thermal protection for HSV-2 and HSV-2 TK^−^.

### HSV-2 TK^−^ thermostabilized in PT film retains efficacy at 40 °C for 8 weeks

**Figure 2:**
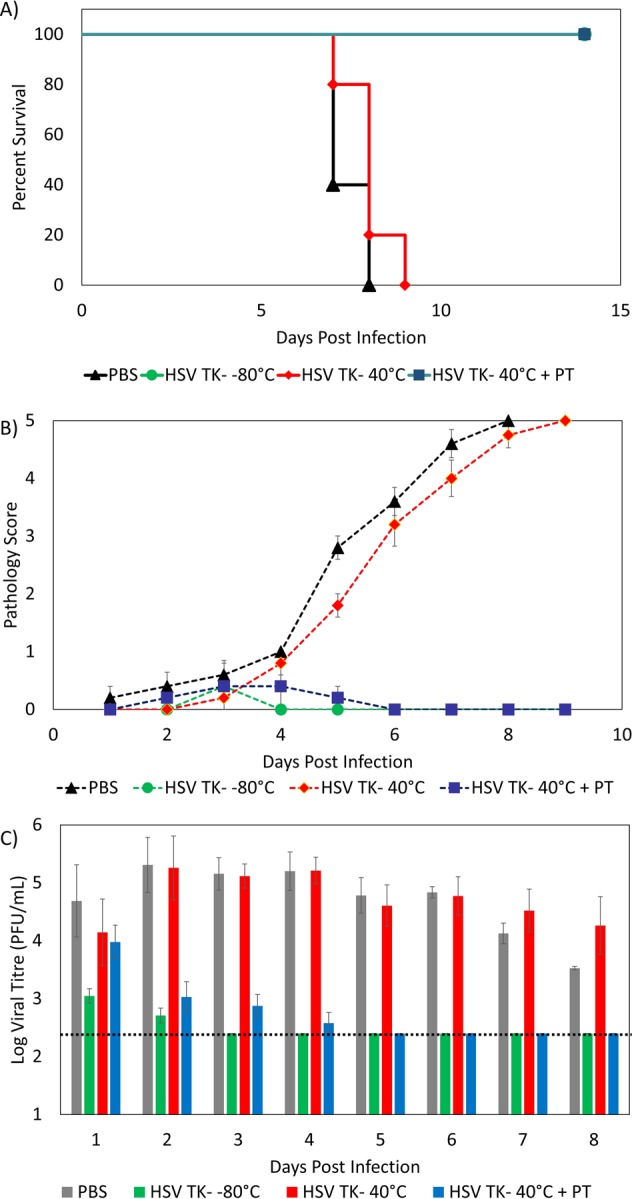
(**A**) Survival curve of mice immunized (i) PBS; (ii) TK^−^ stored at −80 °C; (iii) TK^−^ stored at 40 °C for 8 weeks; (iv) TK^−^ in PT film stored at 40 °C for 8 weeks. Five mice were used for each group. Log rank test was performed to compare the survival of different groups. (**B**) Pathology scores of mice after infection with HSV-2. Error bars indicate the standard error (n = 5). The pathology scores are explained in Methods. Student’s t test was performed to compare the pathology scores of each group. (**C**) Viral titer of vaginal washes as a function of days post infection. Error bars indicate the standard error (n = 5). Dashed line represents the limit of detection. Student’s t test was performed to compare the viral titers of each group.

Having demonstrating the ability of the PT film to thermally stabilize HSV-2 TK^−^ *in vitro*, a subsequent *in vivo* experiment was conducted to determine whether the immunogenicity of HSV-2 TK^−^ had been preserved. C57BL/6 mice were immunized intra-vaginally using the following matrices: (1) HSV-2 TK^−^ stored at −80 °C; (2) PBS; (3) HSV-2 TK^−^ stored at 40 °C for 8 weeks; and (4) HSV-2 TK^−^ dried in PT stored at 40 °C for 8 weeks. 20 mice were immunized in total, with each matrix being used to immunize groups of 5 mice. Since the *in vitro* results show that the titer in the PT films decreased over time, the samples were prepared with a higher initial dose than the therapeutic dose. Each sample had an initial dose of 10^6^ PFU, whereas the therapeutic dose consisted of 10^5^ PFU^44^. For this study, the mice were inoculated with HSV-2 TK^−^ before being exposed to a lethal dose of H SV-2 14 days later. As can be seen in the survival curve in Fig. 2(A), all of the mice that had been treated with the PT-film-preserved HSV-2 TK^−^ vaccine survived the infection, which confirms that the vaccine had retained its efficacy after 8 weeks of storage at 40 °C. Indeed, four of the five mice that had been inoculated with the vaccine that had been preserved in PT film showed no visible signs of vaginal pathology, with only one exhibiting minor signs of infection prior to recovering (Fig. 2(B)). Furthermore, as shown in Fig. 2(C), the viral titer of the vaginal washes correlated well with the pathology data. The mice treated with TK^−^ in PT film resolved their infection within 5 days, with no subsequent detection of viral titer from the vaginal washes. These results show no statistical difference to those of the mice that had been immunized with fresh TK^−^, which all survived and resolved their infections within 3 days. Moreover, mice immunized with TK^−^ in PT films performed significantly better (p < 0.01) than mice treated with TK^−^ stored at 40 °C. The mice immunized with TK^−^ that had been stored at 40 °C all showed signs of severe vaginal pathology and reached their clinical endpoint within 9 days. This result was statistically similar to those obtained for the mice that had been immunized with PBS (placebo): these mice also all showed severe vaginal pathology and reached their endpoint within 8 days. In total, the results of these tests clearly show that PT films are capable of maintaining the efficacy of live-attenuated HSV-2 TK^−^ despite prolonged exposure to elevated temperatures.

### PT films thermally stabilize live IAV *in vitro* and retains infectivity *in vivo*

**Figure 3:**
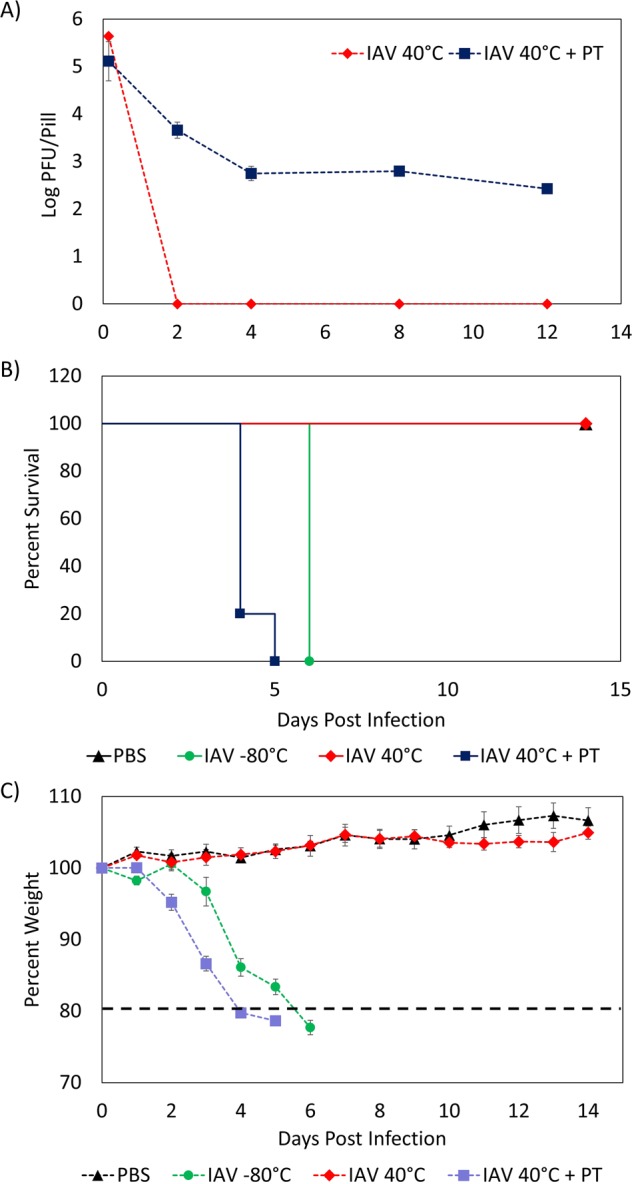
Thermal Stability of IAV in PT Films *in vitro* and *in vivo*. (**A**) Titer of IAV and IAV + PT versus storage time at 40 °C. Experiments were performed in duplicate. Error bars indicate standard deviation. **(B)** Survival curve of mice infected with: (i) PBS; (ii) IAV stored at −80 °C; (iii) IAV stored at 40 °C for 12 weeks; (iv) IAV in PT film stored at 40 °C for 12 weeks. Five mice were used for each group. Log rank test was performed to compare the survival of different groups. **(C)** Weight loss curve post infection.

The results from the HSV-2 experiments demonstrated that PT films are highly capable of thermally stabilizing vaccines for DNA viruses. However, further investigation was needed to determine how suitable PT films are for stabilizing vaccines for RNA viruses. Therefore, we dried an IAV (A/Puerto Rico/8/1934 H1N1, PR8) in 10 wt% pullulan and 0.5 M trehalose and stored the samples for 12 weeks at 40 °C. The titers of the samples were determined at different storage times and compared to those of IAV samples that were stored at 40 °C. As Fig. 3(A) shows, the IAV samples stored at 40 °C became inactive within 14 days, while the IAV samples stored in PT only had a titer loss of 2.0 log PFU/film after the same time period. Much like what was observed in the tests with HSV-2, the IAV samples that had been dried in PT films showed a gradual loss of titer following an initial period of rapid loss. After 4 weeks of storage at 40 °C, these samples showed a titer loss of 2.9 log PFU/film; however, this loss decreased to 0.3 log PFU/film from Week 4 to Week 12. Thus, the total titer loss for the IAV samples preserved in PT film was 3.2 log PFU/film after 12 weeks of storage at 40 °C. Although the *in vitro* results showed that PT films were able to offer some thermal protection for IAV, it also proved to be less thermally stable than HSV-2, as significant titer loss was observed within the first 4 weeks.

Next, the infectivity of IAV dried in PT films was tested *in vivo* by intranasally infecting BALB/c mice using 4 matrices: (1) IAV stored at −80 °C; (2) PBS; (3) IAV stored at 40 °C for 12 weeks; and (4) IAV dried in PT and stored at 40 °C for 12 weeks. The initial dose of the samples was 10^5^ PFU/mouse. Fig. 3(B,C) show that the mice infected with IAV dried in PT film responded similarly to those infected with fresh IAV. Both groups of mice exhibited significant weight loss, with the mice infected with IAV in PT film reaching their clinical endpoint within 5 days, and the mice infected with fresh IAV reaching their clinical endpoint within 6 days. In contrast, the mice infected with IAV that had been stored at 40 °C did not show any weight loss or clinical signs of infection. This demonstrates that storing IAV in PT films retains infectivity, even after 12 weeks of storage at 40 °C. Conversely, when IAV was stored without PT, it became completely inactivated and the mice displayed no clinical signs of infection after the same time period.

### Inactivated IAV vaccine thermostabilized in PT film retain immmunogenicity at 40 °C for 12 weeks

**Figure 4:**
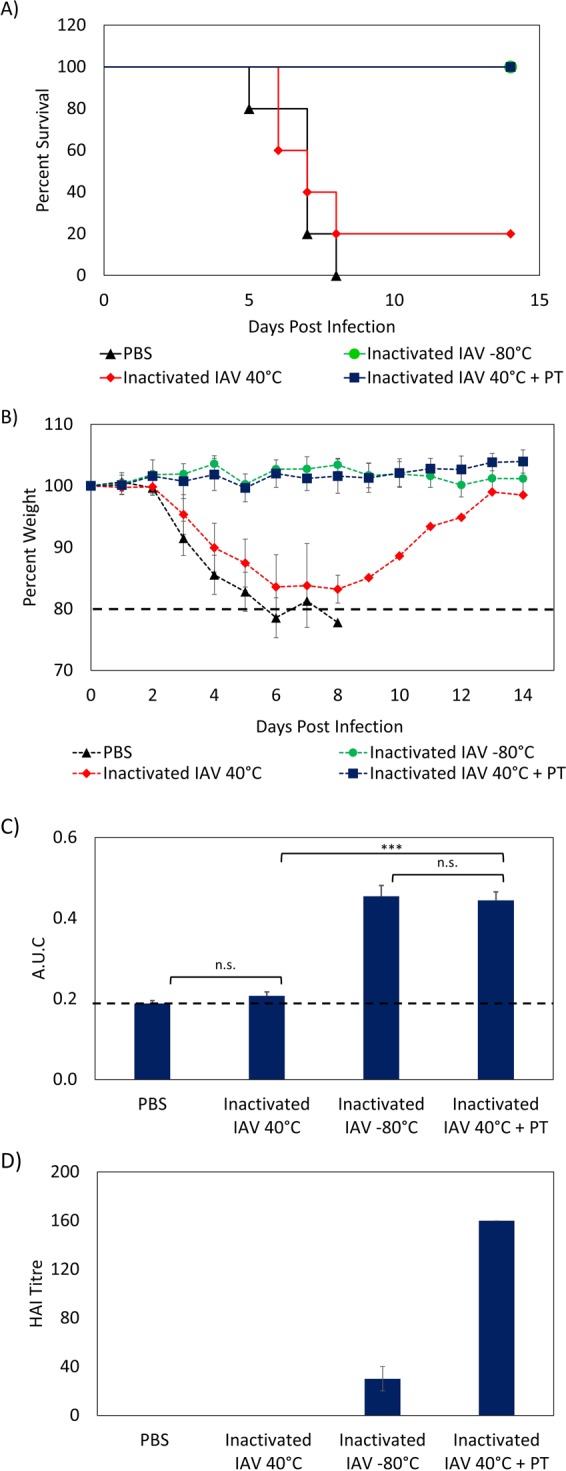
*In vivo* results of mice immunized with: (i) PBS; (ii) inactivated IAV stored at −80 °C; (iii) inactivated IAV stored at 40 °C for 12 weeks; (iv) inactivated IAV in PT film stored at 40 °C for 12 weeks. (**A**) Survival curve. Five mice were used for each group. Log rank test was performed to compare the survival of different groups. (**B**) Weight loss curve post infection. (**C**) Area under curve of ELISA assay for flu-specific IgG for serum samples. Dashed line indicates the limit of detection. Error bars represent the standard error (n = 5). Data were analyzed using one-way repeated-measures ANOVA. Tukey’s multiple comparison test was used to evaluate the statistical differences between means. Statistical significance is indicated as ***(p < 0.001) or n.s. (not significant) indicates not significant. (**D**) HAI titer from HAI assay for serum samples. The assay was performed in duplicate and error bars represent the variation between the duplicate.

The above experiments demonstrate that PT films are capable of thermally stabilizing live viruses (HSV-2 and IAV) while also maintaining their infectivity or efficacy *in vivo*. Given our positive results, we next sought to determine PT films’ ability to thermally stabilize inactivated viral vaccines. In order to do so, we dried formalin-inactivated IAV in PT films and stored them at 40 °C for 12 week. After 12 weeks had passed, BALB/c mice were immunized with 1 of 4 matrices: (1) fresh vaccine (stored at −80 °C); (2) PBS; (3) inactivated IAV stored at 40 °C for 12 weeks; and (4) inactivated IAV dried in PT stored at 40 °C for 12 weeks. The initial dose of each sample contained twice the therapeutic amount in order to account for loss in activity during the drying process and storage. At 30 days post-vaccination, the mice were challenged with 250 PFU of IAV per mouse. Significantly, the mice that had been immunized with the vaccine in PT film stored at 40 °C did not show any statistical difference when compared to the mice that had been immunized with fresh vaccine. All mice in both groups survived 14 days after infection (Fig. 4(A)) and did not exhibit any weight loss (Fig. 4(B)). In contrast, four of the five mice that had been vaccinated with the vaccine stored at 40 °C reached clinical endpoint (>20% weight loss) within 7 days post infection. The one mouse that did not reach endpoint still experienced significant weight loss (>15%) before recovering. The mice that were given the placebo (PBS) all reached clinical endpoint 8 days after being infected. Overall, the survival results shows that mice immunized with vaccine in PT film stored at 40 °C performed significantly better (p < 0.05) than vaccine stored at 40 °C. Furthermore, there was no statistical difference between the mice immunized with vaccine stored at 40 °C and the mice that were immunized with placebo (PBS).

To further investigate the immunogenicity of the vaccines, blood samples were taken from the mice 14 days after immunization to determine antibody titers induced by the vaccine. Total serum IgG and IAV-specific IgG were quantified using ELISA, which revealed significantly higher levels (p < 0.001) of IAV-specific IgG in the mice that had been immunized with the vaccine in PT film than in the mice that had been immunized with the vaccine stored at 40 °C (Fig. 4(C)). Furthermore, there were no significant differences in the levels of flu-specific IgG between the mice that had been immunized with fresh vaccine and those that had been immunized with vaccine in PT film. These results clearly show that the vaccine dried in PT film was able to induce the production of flu-specific antibodies in mice and provide protection against infection, even after being stored at 40 °C for 12 weeks. In contrast, after 12 weeks of storage at 40 °C, the vaccine without PT did not exhibit any immunogenicity, as these mice exhibited the same levels of flu-specific IgG as those that had been immunized with PBS. This result was further confirmed via a hemagglutination inhibition (HAI) assay, which showed an HAI titer of 160 for the mice that had been immunized with vaccine dried in PT and an HAI titer of 30 for those that had been immunized with fresh vaccine (Fig. 4(D)). On the other hand, the mice that had been immunized with the vaccine stored at 40 °C and the mice that had been immunized with PBS both had an HAI titer of 0. The HAI assay results were of particular interest because they showed that the vaccine in PT was capable of generating a greater titers of neutralizing antibodies in mice than the fresh vaccine. While it is possible that this result is the product of using double the therapeutic dose for the initial dose of the vaccine in PT film, it nevertheless demonstrates that inactivated IAV in PT films exhibits excellent thermal stability and vaccine potency for 12 weeks at 40 °C.

## Discussion

The development of thermally stable vaccines is a crucial step in achieving the goal of universal access to immunization. This study presents a simple, cost-effective platform for creating thermally stable vaccines. The results of the research detailed in this paper demonstrate that drying vaccines in pullulan and trehalose, which are both inexpensive and FDA approved, significantly extends the shelf-life of vaccines outside of the cold chain. Specifically, this research shows that the proposed method can effectively be used to thermally stabilize DNA viruses (HSV-2), RNA viruses (IAV), live-attenuated vaccines (HSV-2 TK^−^), and inactivated viral vaccines (formalin-inactivated IAV) for up to 12 weeks at 40 °C. Moreover, our results show that PT films do not interfere with the *in vivo* infectivity, immunogenicity or efficacy of viruses and vaccines.

The *in vitro* experiments demonstrated that pullulan and trehalose together offer better thermal stability than either does on their own. As was observed, pullulan failed to protect the viruses during desiccation when it was used on its own, and trehalose offered poor long-term stability when it was used on its own. One possible explanation for the synergistic behavior between these two compounds may be that trehalose provides protection during desiccation while pullulan offers long-term stability by immobilizing the viruses in a glassy matrix. This explanation would be consistent with previously published results, which note trehalose’s common usage as a desiccation protectant that is often used during lyophilisation^21,29,45^. Moreover, we have previously shown that enzyme mobility is restricted in a pullulan film, even at 60 °C^41^. The restriction of mobility afforded by the pullulan glass matrix results in enhanced thermal stability. Furthermore, pullulan films are oxygen impermeable, which allows them to offer protection from oxidative degradation. Therefore, the synergistic nature of pullulan and trehalose may be attributed to the combination of desiccation protection from trehalose and immobilization within the pullulan sugar glass.

The *in vitro* experiments also showed that, while viral titer significantly decreases within the first 4 weeks of storage, this loss remains relatively stable between the 4 and 12 week marks. This may suggests that there are multiple degradation mechanisms within the PT films. Further investigation into the degradation of viruses within the PT film may help to improve thermal stability by limiting the initial loss of viral titer. One drawback of using PT films as a stabilization method is that, although the rate of titer loss for samples stored in PT films is much lower than in samples stored in solution, titer loss still occurs over time. Consequently, it is necessary to use higher initial doses in order to compensate for this activity loss. One solution to this problem is to optimize the formulation, preparation, and storage conditions of the PT films, a process which we have documented in a previous study wherein we demonstrated how PT films can be used to improve the long-term stability of bacteriophages^42^.

Despite the titer loss for live viruses in PT films, there was no observable loss in efficacy for inactivated IAV viral vaccines. This was likely due to the fact that the inactivated viral vaccine only required the preservation of the antigenic proteins rather than infectivity of the virus itself. Thus, although PT films may need to be further optimized for live-attenuated vaccines, the *in vivo* results showed that they can afford long-term stability for inactivated vaccines without further optimization. Moreover, since previous studies have shown that pullulan and trehalose films are capable of providing thermo-stability for other labile biomolecules, we suggest extending research on this technology to other types of vaccines, such as recombinant or conjugate vaccines.

In addition to thermal stabilization, another benefit of drying vaccines in PT film is the ease of administration it affords for certain applications. Since pullulan is a water-soluble film-forming agent, vaccines dried in PT film do not require reconstitution and can be directly administered in a film format. This is especially useful for vaccines that enter the body through mucosal surfaces, such as those that are compatible with oral or vaginal administration. Furthermore, along with being easier to administer, the use of vaccine films can also minimize storage space requirements.

Overall, we have shown a simple and versatile method for thermally protecting viral vectors using pullulan and trehalose films. This technology has the potential to solve the cold chain problem and in turn greatly improving global health by providing people around the world with access to vaccines.

## Methods

### Pullulan and trehalose pill preparation, storage, and reconstitution

**Figure 5:**
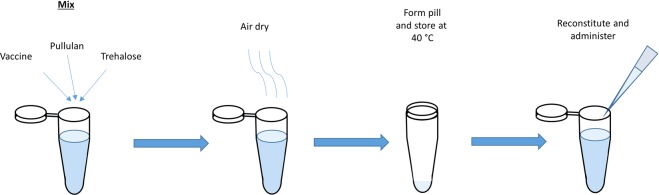
The preparation, storage, and reconstitution of vaccines dried in pullulan and trehalose pills.

Pills containing HSV-2, HSV-2 TK^−^, or IAV were prepared by mixing 1 μL of solution containing the virus with 9 μL of a solution containing 10 wt% pullulan (Polysciences, 200 kDa) and 0.5 M trehalose (Sigma). For the inactivated IAV experiments, IAV was inactivated in chorioallantoic fluid prior to purification. Then 50 μL of the inactivated virus was mixed with 50 μL of 10 wt% pullulan and 0.5 M trehalose and then air dried in a 1.7 mL microcentrifuge tube overnight in a biological safety cabinet. After drying, the tubes were capped and wrapped in paraffin film before being placed in a heating block, where they were stored at 40 °C for up to 3 months. At each time point, a sample was removed from the heating block and reconstituted in PBS. The reconstituted sample was then used for titration for *in vitro* study or administered to a mouse for *in vivo* study. Figure 5 shows the schematic of the process for pill preparation, storage, and reconstitution.

### Cells and viruses

HSV-2 strain 333 was grown and titered as previously described^46^. Vero cell lines (ATCC, Manassas, VA) were maintained in alpha minimum essential medium (α-MEM) supplemented with 1% L-glutamine, 1% penicillin/streptomycin (P/S) and 5% fetal bovine serum (FBS) (Invitrogen, Burlington, ON). Madin-Darby Canine Kidney (MDCK) cells were purchased from the ATCC and grown in Dulbecco modified Eagle medium (DMEM) (Thermo Fisher Scientific) supplemented with 10% fetal bovine serum (FBS) (Gibco), 100 U/mL of penicillin and streptomycin (Gibco), and 2mM L-glutamine (Gibco) at 37 °C with 5% CO_2_. Influenza A virus (A/PR8/1934 [H1N1]) was propagated in 10 day old embryonated chicken eggs according to standard operating procedure.

### *In-vitro* HSV-2 viral titration

Vero cells were grown in a monolayer to confluence in 12- well plates containing α-MEM that had been supplemented with 1% P/S, 1% L-glutamine, and 1% HEPES. The samples were re-suspended and serially diluted (10^−1^ to 10^−6^) in PBS and then incubated with the monolayer for 2 h at 37 °C. Following incubation, the vero cells were overlaid with α-MEM that had been supplemented with 0.05% human immune serum before being incubated for an additional 48 h at 37 °C. Next, the cells were fixed and stained with crystal violet, and the plaques were quantified using a light microscope. The PFU per pill was calculated using the plaque count and the corresponding dilution factor.

### Genital HSV-2 immunization and infection

C57BL/6 (B6) mice aged 6–8 weeks were purchased from Charles River for use in the HSV-2 studies. The mice were housed at McMaster’s Central Animal Facility (CAF) in pathogen–free conditions with a 12-h day/night cycle. All experiments were performed in accordance with Canadian Council on Animal Care guidelines and approved by the Animal Research Ethics Board at McMaster University. For the HSV-2 infection study, the mice were injected subcutaneously with 2 mg Depo-Provera (medroxyprogesterone acetate) 5 days before HSV-2 infection. The mice were then infected intravaginally with 10 μL of the reconstituted HSV-2 sample. For the TK^−^ HSV-2 immunization experiments, the mice were injected subcutaneously with 2 mg Depo-Provera (medroxyprogesterone acetate) 5 days prior to being immunized with 10 μL of the reconstituted HSV-2 TK^−^. The mice were injected subcutaneously with 2 mg Depo-Provera 9 days after immunization and infected with HSV-2 (strain 333) at a dose of 10^5^ PFU per mouse 14 days after immunization. For both the infection study and immunization study, the mice were assessed for genital pathology and survival using the procedure previously described^47–49^. In short, genital pathology was scored on a scale of 5 according to severity of redness, swelling, lesion development, hair loss, ulceration, and lower limb paralysis. The ulceration of a lesion and/or lower limb paralysis was considered to be the clinical endpoint.

### Influenza virus infection

6–8 week old BALB/c mice (Charles River Laboratories, Inc., Wilmington, MA, USA) received either PBS, Influenza A Virus A/PR/8/1934 H1N1 (PR8) (initial titer 10^5^ PFU) that had been stored for 12 weeks at 40 °C, IAV (initial titer 10^5^ PFU) in combination with PT stored for 12 weeks at 40 °C, or 250 PFU IAV that had been stored at −80 °C. The mice were anesthetised with isoflourane and inoculated with 20 uL per nostril, for a total volume of 40 uL per mouse. The weight of each mouse was monitored for 14 days as a measure of morbidity. Weight loss and survival were analyzed using GraphPad Prism 7 (GraphPad Software, La Jolla, CA, USA). Mice were euthanized after losing 20% of their initial body weight. All animal procedures were approved by the Animal Research Ethics Board at McMaster University.

### Influenza virus vaccination

6–8 week old BALB/c mice (Charles River Laboratories, Inc., Wilmington, MA, USA) were vaccinated *i*.*m*. in the left hind limb with one of the following matrices: PBS; formalin-inactivated IAV that had been stored for 12 weeks at 40 °C; formalin-inactivated IAV dried in PT that had been stored for 12 weeks at 40 °C; or formalin-inactivated IAV that had been stored at −80 °C. All vaccinations were administered in 100 uL volumes. 14 days after being vaccinated, the mice were bled via the facial vein and the collected blood was stored at 4 °C overnight. Following overnight incubation, the blood samples were centrifuged at 16,000 × g for 10 min at 4 °C in order to separate the serum. At 30 days post-vaccination, the mice were challenged with 250 PFU of IAV per mouse (as described above) and monitored for weight loss as a measure of morbidity. Weight loss and survival were analyzed using GraphPad Prism 7 (GraphPad Software, La Jolla, CA, USA), and mice were euthanized after losing of 20% of their initial body weight. All animal procedures were approved by the Animal Research Ethics Board at McMaster University.

### ELISA

Enzyme-linked immunosorbent assays (ELISA) were performed in 96-well plates (ThermoFisher Scientific, Mississauga, ON, CA). Each plate was coated with IgG capture antibody (ThermoFisher Scientific, Mississauga, ON, CA) or formalin-inactivated IAV at 2 μg/mL for 24 hours at 4 °C in bicarbonate/carbonate coating buffer (0.05 M Na_2_CO_3_, 0.05 M NaHCO_3_, pH 9.4). The plates were then blocked using 100 μL of 5% non-fat milk in PBS with 0.1% tween (PBS-T) for 1 hour at room temperature (RT). Following blocking, the serum samples were added at starting dilutions of 1:800 in blocking buffer for the IgG wells, and 1:50 for whole-inactivated virus-containing wells. The serum samples were then diluted 1:2 across the plate 11 times, leaving the last well as a blank control. Next, the samples were incubated for 1 hour at RT and then washed 3 times with PBS-T. After washing, 100 uL of IgG-HRP (Santa Cruz Biotechnology, Inc., Dallas, TX, USA) was added at 0.1 μg/mL, before being diluted in PBS-T and incubated at room temperature for 1 hour. Following the incubation period, the plates were washed 3 times with PBS-T. Once washed, 100 μL of Sigmafast OPD substrate (MilliporeSigma, Oakville, ON, CA) was added for 10 minutes before stopping the reaction with 50 μL of 3 M HCl. The plates were then analyzed on the Spectramax i3 plate reader (Molecular Devices, Sunnyvale, CA, USA) at an absorbance of 490 nm, and the resultant data was analyzed using GraphPad Prism 7 (GraphPad Software, La Jolla, CA, USA). Following analysis, the data was transformed into a log(X) scale and a nonlinear fit was performed using the log (agonist) vs. response with a variable slope (four parameters). The area under the curve (A.U.C.) was then graphed, and statistical analysis was performed using a one-way ANOVA with a Tukey post-hoc test.

### Influenza virus quantification

IAV viral titers were determined by plaque assay on Madin-Darby Canine Kidney (MDCK) cells and analyzed using GraphPad Prism 7 (GraphPad Software, La Jolla, CA, USA).

### Hemagglutinin inhibition (HAI) assay

HAI assays were performed as described previously^50^. Prior to performing the HAI assay, serum was pooled and subsequently inactivated. 0.5 volumes of 8 mg/mL TPCK-treated trypsin (MilliporeSigma, Oakville, ON, CA) were added to 1 volume of serum and incubated at 56 °C for 30 minutes. Following incubation, 3 volumes of 0.011 M metapotassium periodate (MilliporeSigma, Oakville, ON, CA) solution per volume of serum was added and incubated for 15 min at RT. After incubation, 3 volumes of 1% glycerol saline solution were added and incubated at RT for another 15 minutes. Finally, 2.5 volumes of 0.85% saline were added to the serum. The inactivated serum samples were serially (2-fold) diluted across a 96-well plate (Fisher Scientific, Ottawa, ON, CA) at 25 uL/well. In addition, 4 HA units of virus was added to all of the wells (25 uL/well), which were then incubated for 30 mins at RT to allow for antibody-virus neutralization. V-bottom plates were used for the HAI assays. Next, 0.5% chicken red blood cells (Canadian Food Inspection Agency [CFIA], Nepean, ON, CA) was added to each well at 50 uL/well. The plate was then incubated at 4 °C for 45 minutes.

### Statistical analyses

Differences in vaginal wash titers and pathology scores were compared using Student’s *t*-test. Repeated-measures one-way ANOVA analyses and the Tukey’s repeated-measures test were performed for evaluation of IgG end-point titers. The survival of different groups were compared by the log rank test.
